# Advances in biomaterial production from animal derived waste

**DOI:** 10.1080/21655979.2021.1982321

**Published:** 2021-11-23

**Authors:** Ayon Tarafdar, Vivek Kumar Gaur, Neha Rawat, Pratik Ramesh Wankhade, Gyanendra Kumar Gaur, Mukesh Kumar Awasthi, Narashans Alok Sagar, Ranjna Sirohi

**Affiliations:** a Livestock Production and Management Section, ICAR-Indian Veterinary Research Institute, Bareilly, Uttar Pradesh, India; b Environment Toxicology Division, CSIR-Indian Institute of Toxicology Research, Lucknow, India; c Department of Food Science and Technology, College of Agriculture, G. B. Pant University of Agriculture and Technology, Pantnagar, Uttarakhand, India; d College of Natural Resources and Environment, Northwest A&f University, Yangling, Shaanxi Province, China; e Division of Livestock Products Technology, ICAR-Indian Veterinary Research Institute, Bareilly, Uttar Pradesh, India; f Department of Chemical and Biological Engineering, Korea University, Seoul, South Korea

**Keywords:** Cattle waste, goat waste, pig waste, poultry, sheep, slaughter house waste

## Abstract

Animal derived waste, if not disposed properly, could pose a threat to the environment and its inhabitants. Recent advancements in biotechnological and biomedical interventions have enabled us to bioengineer these valuable waste substrates into biomaterials with diversified applications. Rearing and processing of poultry, cattle, sheep, goat, pig, and slaughterhouse waste can aid in effective waste valorization for the fabrication of biopolymers, composites, heart valves, collagen, scaffolds, pigments and lipids, among other industrially important biomaterials. Feathers and eggshell waste from the poultry industry can be used for producing keratinous proteins and biocomposites, respectively. Cattle dung, hoofs and cattle hide can be used for producing hydroxyapatite for developing scaffolds and drug delivery systems. Porcine derived collagen can be used for developing skin grafts, while porcine urinary bladder has antiangiogenic, neurotrophic, tumor-suppressive and wound healing properties. Sheep teeth can be used for the production of low-cost hydroxyapatite while goat tissue is still underutilized and requires more in-depth investigation. However, hydrolyzed tannery fleshings show promising potential for antioxidant rich animal feed production. In this review, the recent developments in the production and application of biomaterials from animal waste have been critically analyzed. Standardized protocols for biomaterial synthesis on a pilot scale, and government policy framework for establishing an animal waste supply chain for end users seem to be lacking and require urgent attention. Moreover, circular bioeconomy concepts for animal derived biomaterial production need to be developed for creating a sustainable system.

## Graphical abstract

**Figure:**
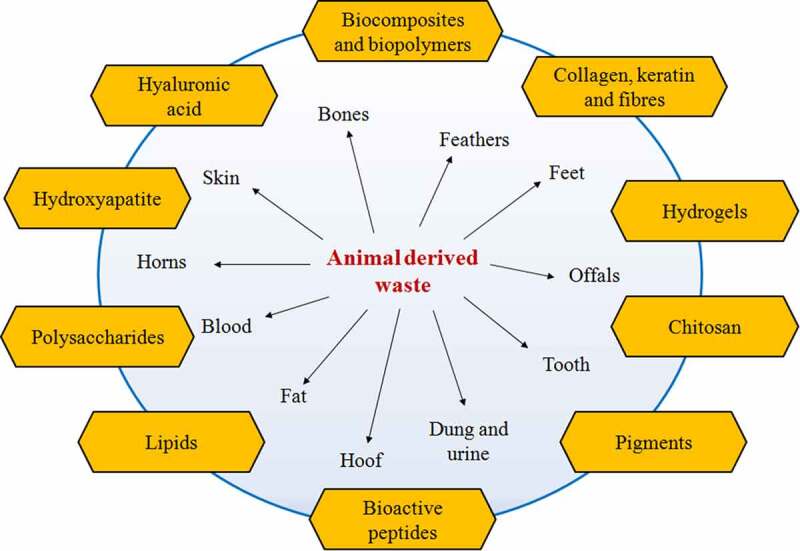

## 1. Introduction

**Figure 1.**
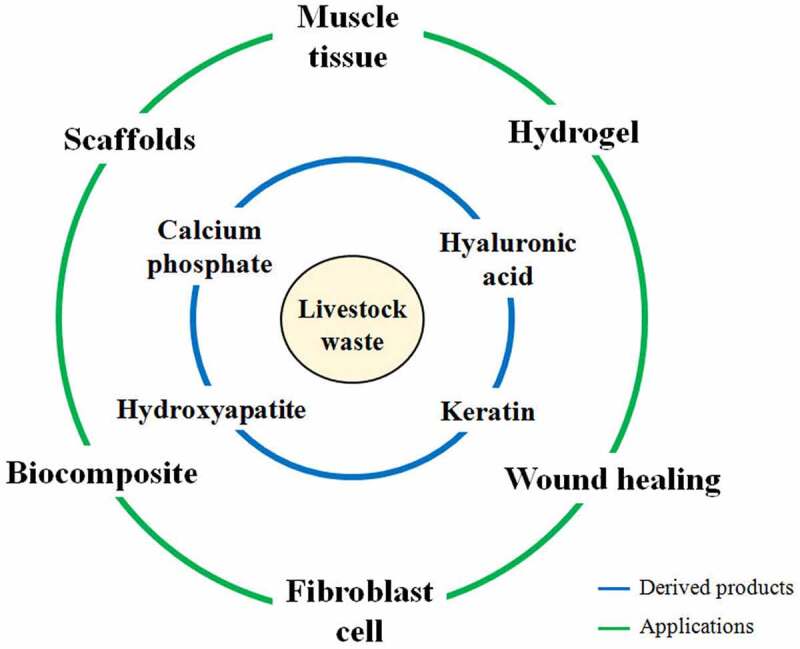
**A**nimal derived biomaterials and applications thereof

Global livestock population is around 4.89 billion including bovines, caprines, ovines, and swine species with around 27.88 billion poultry species [1]. This large number of livestock and poultry, worldwide, produces huge amounts of waste in the order of a few million metric tonnes [1] and are ending up in the environment without any scientific intervention, producing socio-economic and environmental concerns. Due to this reason, appropriate waste management practices are required to properly utilize these potential waste reserves. Animal derived waste could include a mixture of manure and urine, leftover feed and fodders, and carcass waste/offals (Figure 1). With advancement in the animal production practices, alternative uses and treatment of livestock wastes to recover fertilizer, feed, and biopolymers along with significant reduction in pollution have been achieved. However, the production of biomaterials can pave the way for a more efficient method for management of animal-derived waste with generation of advanced biomedical and pharmaceutical applications.

The traditional methods of waste management deal with composting, vermicomposting, biogas production, and value-added product formation that have been extensively investigated [2,3]. However, very scarce investigations focus on the wholesome utilization of other classes of animal waste (offals, skin, bones, hoofs, etc.,]. Researchers across the world are searching for new, innovative, and eco-friendly technologies for animal waste management. Some of the advances include preparation of scaffold, among other materials. Hydroxyapatite, calcium phosphate, hyaluronic acid, keratin, and collagen are some of the most important materials that are derived from livestock waste [4]. Eggshells have also been explored for the production of calcium citrate nanosheets after grinding and treatment with organic acid, which can be used as bone graft substitutes [5]. In addition, biological methods are more reliable for waste management, as they recycle the various constituents of waste into valuable end products while providing a green and cost-effective method of synthesis. Thus, the utilization of animal waste for the fabrication of biomaterials offers an effective strategy that simultaneously aids in reducing environmental pollution.

To our knowledge, there is no available review that provides an account of the biomaterials that can be produced from different categories of animal waste. Therefore, this review focuses on the production of various biomaterials with animal waste as initial substrates for various biomedical applications.

## 2. Biomaterials from poultry-derived waste

Globally, poultry slaughter industry introduces 7 billion tonnes of feather waste annually to the solid waste biomass. It was reported that the feathers contained >90% keratinous protein and may serve as a low cost and abundant protein source [6]. Various ionic solutions, enzymes, and chemicals have been used to derive the functional keratin from this waste. Since the properties of keratin primarily depend on the extraction method, it has been extensively investigated [7,8].

Recently, Oluba et al. fabricated a biocomposite film by utilizing ginger starch in combination with chicken feather derived keratin [8]. The increase in the concentration of chicken feather keratin significantly increased the tensile strength and surface smoothness whereas the solubility and transparency of the biocomposite film decreased. It was recorded that keratin addition improves the mechanical properties and stability in water, suggesting its use in industrial applications [8]. Furthermore, the chicken feather derived keratin (weight; 20 kDa) exhibiting 14–15% residue of cysteine, was used with glycerol to synthesize keratin film [9]. Glycerol was used as a plasticizing compound to overcome the brittle nature of protein film. It was found that glycerol mixed at a concentration of 0.3 g/g keratin showed good tensioactive properties such as elongation (111.37%), strength (7.56 MPa) and Young’s modulus (27.61 MPa). The increase in glycerol concentration from 0.3 to 0.5 g/g keratin decreased the Young’s modulus to 10.88 MPa. This suggested that glycerol positively affects the flexibility of biofilm. The films had an average thickness of 0.15–0.25 mm and were loaded with rhodamine B to study drug release rate. It was recorded that initially after a burst release of rhodamine B, a continuous release was seen upto 12 h. The release of drug was found to be influenced by change in pH. For instance, 94% of drug was released in 12 h at 7.5 pH whereas only 40% of rhodamine B was released at 3.6 pH [9]. In another investigation, the keratin from chicken feather was used to produce wound dressings and three different combinations viz., keratin (CFK), keratin-chitosan (CFK-C), and keratin-sodium alginate (CFK-SA) were tested [10]. It was found that CFK-C and CFK-SA showed enhanced antibacterial potential against Gram stain positive and negative bacterial strain. The fabricated material CFK-C and CFK-SA exhibited strong cytocompatibility and good cell viability. Furthermore, 100% healing was recorded in 23, 21, 17, and 15 days for untreated control, CFK, CFK-SA, and CFK-C, respectively and thus it can be utilized as a biomaterial for wound dressing [10]. A polymer hydrogel was prepared by polymerizing feather keratin with itaconic acid and N-isopropyl acrylamide in a two step polymerization process using a crosslinker. This had led to the formation of interpenetrating network with feather keratin. The biopolymer hydrogel exhibited good deswelling and swelling potential with sensitivity toward temperature and pH. The hydrogel was studied for the release of doxorubicin hydrochloride (DH), an anticancer drug, and bovine serum albumin (BSA). A cumulative release of 93.3% DH in 16 h and 75.9% of BSA in 24 h was recorded [11]. A porous scaffold was developed by blending agar and chicken feather derived keratin. The scaffold was prepared by mixing 5% grinded feather and 5% agar solution followed by employing the freeze extraction technique. This led to the formation of an interconnected porous structure with 94.40% porosity and 50–300 nm pore size. The scaffold was hydrophobic with 160% water retention capacity, 16.33% elongation till breakage and 0.154 MPa of tensile strength. Furthermore, good antimicrobial property, cell viability and negative cytotoxicity makes it an ideal candidate to be utilized in tissue engineering applications such as skin regeneration and wound healing [12]. Not only the keratin extracted from chicken feather but the chicken feather fiber itself can be used for the production or fabrication of different biomaterials. In a study, epoxy resin was used as a matrix to fabricate a hybrid biocomposite material by using different weight percent of chicken feather fiber to provide reinforcement. It was recorded that addition of chicken feather fiber reduced the weight density by 800 kg/m^3^ of the composite in comparison to pure epoxy. Furthermore, the increase in the concentration of chicken feather fiber from 0% to 5% reduced 3.79% of the weight density i.e. from 1132.08 kg/m^3^ to 1089.12 kg/m^3^, and a further rise in feather concentration to 7% decreased the density to 1078.51 kg/m^3^ [13]. The reason to this linear decrease in the density was attributed to the commutative addition of chicken feather fibers in epoxy. Furthermore, the tensile and impact strength was found affected by addition of these fibers. The impact strength of epoxy resin was 1230 J/m^2^ whereas upon addition of 7% of chicken feather fiber, the impact strength increased to 1480 J/m^2^. Unlike this, the tensile strength decreased with increasing concentration of chicken feather fiber and the reason for this was the irregular shape and the poor strength of the fibers [15].

Another major waste generated from poultry is the shell of chicken egg. Since chicken egg shell contains 94% of calcium carbonate (CaCO_3_), it can potentially serve as a raw material for the synthesis of biomaterials. The solution of CaCO_3_ when mixed with H_3_PO_4_ and NaHCO_3_ yields carbonated-hydroxyapatite (CHA) in the presence of microwaves. The CHA is also the primary inorganic constituent of bones in humans. Thus, this can be efficiently utilized for the fabrication of scaffolds to be used in bone tissue engineering [15]. It was reported that globally, approximately 77 million ton of hen eggs were produced annually [16]. The morphology and mechanical properties of egg membrane were ideal for its use in the fabrication of biomaterials for wound dressing [17]. Different extraction processes have been used viz. manual peeling, use of ethylenediamine tetraacetic acid (EDTA) or acetic acid for the isolation of eggshell membrane. This membrane shows excellent mechanical properties, optical transparency, porosity, and biocompatibility with corneal mesenchymal stromal cells and immortalized corneal epithelial cells. Thus, it can be utilized as a biomaterial for corneal wound healing [17]. In a study by Jena and Sahoo, bionanocomposite material was prepared by emulsion polymerization using chicken eggshell powder and starch-gpoly(N-isopropylacrylamide) [18]. The addition of eggshell powder significantly improved the tensile strength and Young’s modulus. Thermogravimetric analysis revealed that the biocomposite showed thermal stability and the reason to this was attributed to the enhanced char forming property of eggshell powder [18]. Furthermore, the bionanocomposite derived by addition of 4% w/v eggshell powder showed 33.3% decrease in peak heat release rate and 75.3% decrease in peak smoke production rate and thus served to suppress fire hazards [18].

The organic waste released from poultry industry is rich in collagen. Gronlien et al. Isolated collagen from Turkey was soluble in pepsin [19]. The isolated collagen was found to be biocompatible and thermo stable. The collagen scaffolds containing riboflavin and prilocaine hydrochloride showed a slow release of these drugs and served as a promising biomaterial to be used as drug carrier and other pharmaceutical use [19]. The chicken shank collagen was mixed with chitosan to form a chitosan-collagen scaffold for its possible application in bone regeneration. The scaffold was prepared by homogeneous mixing of chicken shank collagen gel and chitosan gel in two different ratios viz. 20:80 and 50:50. In an *in vivo* assay on Wistar rats (*Rattus norvegicus*), it was recorded that the highest lymphocyte cell proliferation was found in the bone defects treated with chitosan-collagen scaffolds with 50:50 gel mixing as compared to individual collagen and Natrium-Carboxy Methyl Cellulose (3%) [20].

## 3. Biomaterials from cattle derived waste

Among the waste generated by cattle, the excreta and the bones were most studied for their utilization in the fabrication of different products for economic and social value [21–23]. The biocompatible ceramic, namely hydroxyapatite (a primary and essential constituent of teeth and bone) isolated from bovine bones has found various biomedical applications [21–25]. A study conducted by Hilmi et al. showed that hydroxyapatite was obtained by de-fatting of bovine bones followed by a calcination process at 900°C [26]. The produced bovine hydroxyapatitie was highly crystalline with a particle size of <45 µm. In another investigation, the hydroxyapatite from bovine femur bone conjugated with silver nanoparticles was prepared by the process of thermal decomposition and silver nitrate reduction with N,N-dimethylformamide. In this complex, the hydroxyapatite was surrounded by 8–20 nm size silver nanoparticles. The synthesized hydroxyapatite−silver nanoparticle complex exhibited good antibacterial property against methicillin resistant *Staphylococcus aureus* (MRSA), non-MRSA and *Escherichia coli* [25]. Furthermore in 2014, the researchers became interested in developing fluorinated hydroxyapatite derived bioceramic materials. This was driven by the observation that the addition of fluorine ions increased the stability of hydroxyapatite in biological systems and promoted the formation of apatite. Owing to this, Khandan et al. synthesized the fluorinated hydroxyapatite ceramic using 4.3 wt.% calcium fluoride (CaF_2_) powder and 95.7 wt.% natural hydroxyapatite by employing a mechano-chemical method. The synthesized fluorine-hydroxyapatite exhibiting a sphere distribution and had a 80–90 nm crystal size [24]. To improve the function of printed scaffold used in bone regeneration, the hydroxyapatite was mixed with starch to prepare a composite material to be used in robocasting.

Jellification process rapidly solidifies the hydroxyapatite starch composite. The addition of starch to hydroxyapatite guarantees the bimodal size distribution that leads to a decrease in the porosity and improved strength of the jellified product [21]. The hydroxyapatite derived from bone powder can also be fabricated to be utilized in dental implants. The processed bone powder containing particles <250 µm was calcinated to obtain hydroxyapatite. This was then sintered into a block and the contact angle that provides the measure of average wettability/material hydrophobicity was 31.73 degrees. The porous hydroxyapatite was found stable when studied under the human physiological condition. The hydroxyapatite was compatible to the body tissue osseointegration and was not absorbed in the body fluids. Also, increasing the pores in the block reduced the hardness of the scaffold [22].

Furthermore, bovine origin powdered and sintered hydroxyapatite was mixed with type I collagen to develop osteoinductive and osteoconductive scaffolds. The particle size in the scaffold varied between 200 and 400 nm. The surface-to-volume density and the volume density of the composite material varied from 5.090 to 6.366 µm^−1^ and 0.45 to 0.55 µm^−1^, respectively. This small change in the volume density and surface to volume density suggested the macroporous structure of the biocomposite scaffold and, upon further investigation it was recorded that the human osteoblast cells adhered to both the collagen and hydroxyapatite particle [27]. Meat industry produces a significant amount of collagen as waste from short tendons of slaughtered cattle and cattle hides. This collagen can be utilized for the production of various biomaterials for biomedical field such as for the preparation of sponges for wounds, mini-pellet for drug delivery, nanoparticle for delivery of genes, shields used in ophthalmology, support biomaterial for the formation of neo-organs, etc [28]. Another major waste generated was the animal dung. On average, the annual production of 2600 million ton of cow dung poses a serious threat to the environment by contaminating water sources and contributing in greenhouse gas release, if not disposed properly [23]. Cow dung was reported to serve as an ideal raw material for the extraction of cellulose, lignin, and hemicellulose by the process of Kraft pulping. The biomaterial nanocellulose was synthesized from cellulose that exhibited good surface charge and excellent particle stability [23].

## 4. Biomaterials from pig-derived waste

Pig waste based biomaterials have also found several applications in the biomedical industry. There are three major forms of prosthetic mesh. Synthetic meshes with high tensile strength, such as polypropylene (PP) or polyester, are expected to induce bowel adhesions, rendering them inappropriate for intra−abdominal use. Composite meshes, also known as barrier meshes, comprise dual-sided prostheses with a synthetic parietal side facilitating robust healing and a visceral side that resists tissue ingrowth and reduces adhesion development. Biological meshes comprise of collagen-based scaffolds that could be implanted extra− or intra−peritoneally [29]. Biobrane, a porcine collagen-coated nylon net that may be placed topically to the wound and readily removed following re−epithelialization, eliminates the necessity of daily dressing changes [30]. Furthermore, as compared to traditional dressings, it enhances and speeds skin recovery. Temporary skin grafts are recommended in serious burns whenever the patient’s life is at stake by significant skin loss. Human allografts and xenografts produced from pig skin are also used. Biological and mechanical valves are the two types of prosthetic valves. Biological valves, also known as bioprostheses, are made out of human or animal tissue and are divided into autografts, homografts, and heterografts (porcine/bovine derived). The durability of new-generation porcine valves has been reported to be between 10–15years [31]. A dense tissue type (cortical bone) and a spongy porous substance make up the bone (trabecullar bone). A bone lamella, which is generally approximately 5 m thick across both tissue types, is the fundamental building component. Lamellae in cortical bone generate secondary osteons, which are layered cylindrical composite structures formed around blood veins. The mechanical performance of bone, also known as bone quality, is determined not only by the shape and quantity of bone (as measured by BMD), but also by its architecture and the quality of the bone material [32]. When used to replace a damaged component, bone can be used as a biomedical material. The grafting which involves the utilization of the patient’s own bone in the place of the fractured part is called as autografting, whereas allografting is the utilization of another human being’s bone and it often involves the utilization of cadaver. However, utilization of animal bones such as pigs, rabbits, dogs, among others, acts as a replacement and is referred to as xenografting [33]. Bone by weight typically consists of 25% water, 15% organic materials and 60% mineral phases. The mineral phase primarily comprises of calcium and phosphate ions, with some amount of carbonate, magnesium, hydroxyl, fluoride, chloride and citrate ions [34]. In a study, a revised connective tissue graft wall method with enamel matrix derivative has been utilized to repair vertical bony deformities. A coronally advanced flap was used along with a porcine-derived acellular dermal matrix inserted beneath, pretending as the buccal soft tissue wall of the bony defect. The location of the interdental papilla as well as clinical attachment level gain increased one year following the surgery, along with radiographic bone defect fill [35]. In another study, the histologic and ultrastructural properties of a biomaterial made of cortical pig bone granules were assessed. Under light microscopy, it was observed that majority of the particles were enveloped by freshly produced bone. The osteoid matrix was present in some locations, although primarily compact bone was observed at the interface. No signs of an acute inflammatory infiltration were found. The newly produced bone was 36%–2.8% and the marrow gaps were 38%–1.6%, whereas the leftover grafted material was 31%–1.6%. All stages of bone development (osteoid matrix, woven, and lamellar bone) were seen in close proximity to the biomaterial components under TEM. The bone biomaterial interface revealed intimate contact between the porcine bone particles and the surrounding bone, which exhibited mature bone characteristics with many osteocytes [36]. When protein profile of a xenogeneic biomaterial, which was derived from porcine urinary bladder matrix (UBM), was analyzed, about 129 proteins exhibiting antiangiogenic, neurotrophic and tumor-suppressive properties along with tissue remodeling and wound repair properties were recognized [37]. It was also observed that porcine-derived biomaterial assists in human osteoclasts formation [38]. The cytocompatibility of a hydrogel generated from vocal fold lamina propria-extracellular matrix (VFLP-ECM) was investigated, as well as the compositional aspects of decellularized porcine VFLP-ECM scaffold and possible antifibrotic effects. The findings revealed the VFLP-unique ECM’s protein composition, as well as a link between the VFLP-component ECM’s and the reduction of TGF-1 signaling found *in vitro* when turned into injectable forms [39].

## 5. Biomaterials from sheep-derived waste

Hydroxyapatites (HAs) have good biocompatibity and are mostly utilized in bone replacement and tissue engineering [40]. Hydroxyapatites derived from natural sources are considered safer pertaining to their cross-reaction and other immunological reaction in comparison with synthetic hydroxyapatite [41]. Synthetic HA biomaterials are extremely dependable; however, HA production is typically difficult and costly. Bio-ceramics made from naturally occurring biological apatites are less expensive. In a study, sheep teeth dentine HA material was used as an alternate source of bioactive biomaterial for the purpose of grafting. Sheep teeth were extracted, cleaned, and calcinated in air at 850°C. After calcination, the enamel substance was easily separated from the dentine. The dentine fragments were crushed and ground in a ball mill. In order to make samples suited for compression and microhardness testing, the powder was crushed between hardened steel dies. Sintered powder compacts were obtained at various temperatures: 1000, 1100, 1200, and 1300°C in air [42]. The findings of this study revealed that the HA material derived from sheep tooth dentine may be considered a promising source of HA for bioactive ceramics production. HA material can be manufactured synthetically as well as naturally. Synthetic HA requires expensive reagent-grade chemicals along with tedious and time-consuming methods. HA materials can be manufactured using various methods, e.g., calcinations (human HA, bovine HA, sheep HA), chemical synthesis with hot-plating, ultrasonication, or hydrothermal methods [43,44].

## 6. Biomaterials from goat-derived waste

Among various animals used in manufacturing biomaterials, goat tissue is still underutilized in tissue engineering, although it is comparatively less susceptible to contamination or disease transmission than cadaveric porcine and bovine tissue. In a study, small intestinal submucosa (G-SIS) of goat was isolated out of goat’s small intestine (G-SI), which is often a waste obtained from goat-slaughterhouse, and was decellularized in order to obtain decellularized G-SIS (DG-SIS) biomatrix in powder form, gel form and sponge form owing to its potential in healing different types of wounds. Nanoceria (NC) was induced in the DG-SIS in order to fabricate DG-SIS/NC nano bio-composite scaffold, which provides synergistic effects in rapid tissue regeneration. The scaffolds so obtained were hydrophilic, haemo-compatible, biodegradable, antibacterial, biocompatible and revealed free-radical scavenging capability. It was observed that the scaffolds containing higher NC concentration (500 µg ml^−1^) depicted highest cell (fibroblast cells) adhesion, free radical scavenging activity and MTT activity in comparison to the DG-SIS and other nano bio-composite scaffolds. Thus, DG-SIS/NC3 scaffolds can be used as potential scaffold biomaterial for skin TE application. Collagens employed in tissue engineering nowadays are mostly from bovine or porcine sources. The potential of a spongiform encephalopathy epidemic has, however, limited the usage of collagen derived from these sources. Some scientists investigated the prospect of employing domestic goat accessible on the subcontinent as a viable supply of collagen for tissue-engineering applications, keeping the aforementioned perspective in mind. GTC (Goat tissue collagen) was discovered to be made up of type-I collagen. GTC improved cell adhesion, cell cycle progression, and proliferation, according to a biocompatibility study. Immunocytochemical study in combination with traction force microscopy demonstrated that the cell–substrate connection in GTC is mediated by a superior focal adhesion complex. Finally, an *in vivo* investigation in mice indicated that GTC has low immunogenicity and dramatically improves the healing process. Calf skin collagen (CSC) was employed as a benchmark for comparison throughout the investigation. Therefore, GTC has the potential to be used as a biomaterial in skin tissue engineering [45]. Goat hoof can also be regarded as a naturally present composite material made up of tubular and inter-tubular keratin that contributes to simulate the tissue’s extracellular matrix. A medulla (potentially hollow) is encircled by a cortex of keratinized cells in each tubule. Goat hoof keratin (GHK) is a biodegradable material that can be used to regenerate tissue. Using a modified shindai solution process, the protein material from GHK powder is extracted in a clean form. The SDS-PAGE analysis of this protein yielded polypeptide chains with a molecular weight of 40–45 kDa after electrophoresis. It was reported that GHK denaturation occurred in the temperature range of 200°C to 250°C, and thermal deterioration occurred in the range of 211°C to 387°C [46].

Apart from hoof-derived protein powders, protein hydrolyzates obtained from goat tannery fleshings have also been investigated for potential applications in antioxidant rich animal feed. These hydrolyzates after fermentation by *Enterococcus faecium* strains show rich reserves of the amino acids arginine and leucine, indicating their importance in reducing oxidative stress in animals [47]. Moreover, the fermented hydrolyzate shows antagonistic effects against *Salmonella typhi* with 83%, 59%, 88.2% and 85.4% antioxidant activity against 2,2-azino-bis-3-ethyl-benzthiazoline-6-sulfonic acid (ABTS), 2,2-diphenyl-1-picrylhydrazyl (DPPH), peroxy and superoxide anion radicals at different protein hydrolyzate conditions [48].

## 7. Biomaterials from other sources of animal waste

Table 1 presents the summary of biomaterials generated from different sources of animal waste. Apart from those already discussed, there are many other sources of animal waste that can be explored for the production of biomaterials. For instance, polysaccharides, the most important class of biopolymers, are basic materials that can be derived directly from animal by-products. Polyhydroxy alkanoates (PHAs) with large molar percentages of 4-hydroxybutyrate (4HB) are produced by *Delftia acidovorans* DSM39, but it cannot thrive on fatty substrates. A study was conducted to develop a *D. acidovorans* DSM39 recombinant strain capable of producing PHAs from waste fats such as lard, udder and tallow. Interestingly, the recombinant strain was able to obtain 43 and 39%, respectively, with almost 7% of 4HB from udder and fat, respectively. The findings of this study correspond to a one-step conversion of fatty residues into PHAs with beneficial properties that might be used in a wide range of industrial applications [49]. The use of animal fat has been explored for the production of carotenoids (torulene, torularhodin, β-carotene) and lipids by feeding them to oleaginous red yeasts as nutrient source. In a study, mixed animal fat media was used in crude, emulsified, and enzymatically hydrolyzed form for the growth of red yeast strains and the production of carotenoids and lipids. Reports showed that the emulsified and hydrolyzed forms were better utilized by the yeast strains and led to higher generation of value added products [50]. In other studies, purification of waste animal fat by vacuum distillation to generate triglycerides (TAG) has been attempted to produce free fatty acids that were blended with standard diesel at the tune of 40% to satisfactorily run internal combustion engines [51].

**Table 1.** Biopolymers extracted from animal waste and their applications

Source	Waste material	Biopolymer extracted	Applications	References
Poultry	Bone and feathers	Collagen and bioactive peptides	As fillers in polymer industry and tissue engineering	[56,57]
Cattle	Achilles tendon	Bioactive peptides	Anti-obesity, blood pressure and cholesterol-lowering agent in health sector, as active packaging material in food sector	[58,59]
Cattle, pig	bovine lung, skin, bones	Hydrolyzed collagen	Prosthetic heart and collagen sheet or film for organs in health sector	[57]
Cattle	Bovine nuchal ligament	Bioactive peptides	Inhibitory peptides in food the industry	[60]
Pig	Skin	Protein hydrolyzates	Food ingredients, cosmetics and pharmaceuticals	[61]
Poultry	Chicken legs	Hydrolyzed collagen	As and ACE-inhibitor in health sector	[62]
Poultry	Chicken feet	Collagen polymeric fibers	Cosmetic, pharmaceuticals, and biomedical industries	[63]
Cattle	Bovine bone	Bio-Hydroxyapatite	For bone grafting, orthopedic, and dental support	[64]
Ostrich	Waste cortical bones	Bio-Hydroxyapatite	As bone graft material in health sector	[65]
Poultry	Egg shell	Hydroxyapatite	Development of biofilms and bio-ceramics	[40, 66]
]Cattle	Bovine bones	Hydroxyapatite	Biofilm development	[40]

Dairy processing waste is another important resource that contains soluble organics, suspended solids, and trace organics (fats, oils, and grease, minerals, and phosphates), along with other essential components for microbial fermentation to produce biopolymers [52,53]. At pH 7 and temperature of 37°C, with a shaker speed of 150 rpm, buttermilk functions as a carbon source during fermentation, resulting in maximal synthesis of poly 3-hydroxybutyric acid (PHB) [54]. Buttermilk is also the most cost-effective way to make microbial polymer from bacteria isolated from dairy waste. Therefore, dairy wastes are low-cost substrates that provide a green route for the synthesis of biopolymers [55].

The blood from slaughterhouses has also been used to develop biocomposite scaffolds for muscle tissue. The generated scaffold exhibited elastic mechanical properties, a microporous structure, excellent cell proliferation and migration, and good *in vivo* biodegradability [4].

## 8. Conclusions and perspectives

Animal processing and farm wastes contribute to biomaterial development and are generally derived from bones, hoofs, skin, and teeth. The production of biopolymers (PHAs, PHBs etc.), composites, collagen, and other biomaterials from animal wastes, for biomedical and industrial applications was discussed in this review. Currently, biomaterials from animal waste are used for the fabrication of scaffolds, for promoting bone and tissue regeneration and for fibroblast cell growth. However, the method of biomaterial preparation lack standardized protocols in terms of pre-treatment, extraction, chemical modifications and purification that restrict its continuous commercial production. Process viability and optimization is required in this regard that should be achieved through pilot-scale investigations and not in a flask. Important parameters such as the crystallinity, hardness, porosity, surface area, swelling and elasticity, temperature and pH stability, cell viability, cytotoxicity/cytocompatibility, neurovascularization capacity, etc., should be investigated in-depth for the generated biomaterials. Moreover, the diversified production of biomaterials has not been fully explored utilizing animal waste. The comparative merit of biomaterial developed from animal waste should also be explored in contrast to biomaterials produced using other waste sources. Process economics and circular bioeconomy concepts also need to be developed for animal waste-derived biomaterials to make the industrial process of biomaterial fabrication more economical. Further, mere scientific interventions and industrial efforts may not be enough to successfully commercialize the production and distribution process of biomaterials generated from animal-derived waste. There should also be a continuous supply of resources to both research and industrial sectors to produce biomaterials more sustainably. For this, there is a need for government policy framework that would necessitate collecting animal-derived waste from the source and supplying it to the concerned industries and research organizations at subsidized charges. Such frameworks are the need of the hour that would promote both scientific advancement and national economy.

## Data Availability

All the data pertaining to this work is available within the text and no additional data is required.

## Highlights

- Animal waste-based biomaterials are mostly used for tissue engineering applications
- Keratin and collagen from poultry and goat waste are used for biocomposite development
- Teeth and bone from cattle and sheep waste can be used for hydroxyapatite production
- Allografts and xenografts can be produced from porcine waste
- Dairy and slaughterhouse waste are potential resources for biomaterial production
